# Hypothalamic and metabolic alterations at a pre‐symptomatic stage of Alzheimer's Disease

**DOI:** 10.1002/alz70855_103091

**Published:** 2025-12-23

**Authors:** Pablo Valderrama‐Carmona, Federico Pratesi, Paule E.H. M'Bra, Marta Turri, Anne Aumont, Marian Mayhue, Andres Villegas‐Lanau, Rafael Posada‐Duque, Karl JL Fernandes

**Affiliations:** ^1^ Research Center on Aging (CdRV), Sherbrooke, QC, Canada; ^2^ Université de Sherbrooke, Sherbrooke, QC, Canada; ^3^ Université de Montréal, Montréal, QC, Canada; ^4^ Grupo de Neurociencias de Antioquia, Universidad de Antioquia, Medellín, Antioquia, Colombia

## Abstract

**Background:**

The hypothalamus is the brain's center for metabolic regulation. It controls energy homeostasis, adiposity and glucose metabolism, acting as a link between the brain and peripheral tissues.

Alzheimer's Disease (AD) is a multifactorial neurodegenerative disease. Besides the well‐known cognitive impairment, patients often exhibit metabolic disturbances, including insulin resistance, dyslipidemia and body‐weight disorders. Interestingly, these comorbidities have been associated with hypothalamic dysfunction; however, the role of the hypothalamus in AD remains largely unexplored.

In this study, we aimed to characterize early hypothalamic cellular changes and identify potentially associated metabolic alterations during the pre‐symptomatic stage of AD using the 3xTg‐AD mouse model.

**Method:**

To characterize hypothalamic cellular changes at a presymptomatic stage we performed single‐cell RNA sequencing (scRNAseq) on microdissected hypothalami from 3‐months old WT and 3xTg‐AD mice. Bioinformatic analysis were conducted in R.

To gain further insight into metabolic‐related hypothalamic functions we performed body‐weight and blood glucose measurements, analyzed body mass composition and weighted peripheral metabolic organs of 3‐months old 3xTg‐AD mice.

Additionally, we conducted a pilot snRNAseq experiment using post‐mortem human hypothalami from control, familial (FAD) and sporadic (SAD) AD patients (*n* =  1) in collaboration with the Brainbank of the University of Antioquia.

**Result:**

We identified presymptomatic transcriptomic changes in hypothalamic endothelial cells in 3xTg‐AD mice. These changes were primarily observed at the capillary level and were related to immune and stress response.

At 3‐months, 3xTg‐AD mice exhibited sex‐specific metabolic alterations. While females showed significant increases in body weight, glucose levels, fat and pancreas mass, males exhibited significant decreases in the same parameters compared to WT mice.

The pilot snRNAseq experiment with human samples was successful, obtaining ∼10000 nuclei/patient, comprising the different brain cell types, with large populations of neurons and oligodendrocytes identified.

**Conclusion:**

Our results reveal early transcriptomic changes in hypothalamic endothelial cells of 3xTg‐AD mice, related to immune and stress responses. Furthermore, peripheral metabolic alterations were also observed at this stage, suggesting a sex‐specific effect. We propose that hypothalamic dysfunction may drive these metabolic alterations and play a key role in AD. Further analyses are underway to confirm this association using mouse and human samples.

